# Expression of urotensin II is associated with placental autophagy in patients with severe preeclampsia

**DOI:** 10.1038/s41371-018-0083-9

**Published:** 2018-07-10

**Authors:** Ya-Jing Pan, Lian He, Si-Jia Zhou, Li-Jie Zhang, Ai-Hua Zhang, Yang-Yu Zhao

**Affiliations:** 1 Department of Nephrology, Peking University Third Hospital, Beijing, China; 2 Department of Gynaecology and Obstetric, Peking University Third Hospital, Beijing, China

## Abstract

The aims of this study are to explore the correlation between the expressions of urotensin II (UII) and autophagic markers (LC3 and P62) in patients with severe preeclampsia (SPE). A total of 64 pregnant subjects were recruited, including 29 healthy pregnancies and 35 preeclamptic patients (7 mild preeclamptic (MPE) patients and 28 SPE patients). UII and autophagic markers expression in placenta specimens was investigated by immunohistochemistry (IHC), RT-qPCR, and western blot. IHC analysis manifested that the expressions of UII and autophagic markers were mainly located in the placental cytotrophoblast and syncytiotrophoblast. Western blot and IHC analysis both indicated that the expression of UII was significantly correlated with autophagic marker LC3II (by western blot) or LC3 (by IHC) (*r* = 0.495, *P* = 0.010; *r* = 0.816, *P* = 0.007). Moreover, SPE group had higher expression of UII and LC3II, lower expression of P62 than that of normal controls. The expression of LC3II was positively related with systolic blood pressure (SBP) and urinary protein level (SBP (*r* = 0.501, *P* = 0.003) and urine protein quantitation (*r* = 0.509, *P* = 0.022)), whereas P62 had negative correlation with SBP. We first verify that UII has positive correlation with autophagic marker LC3 in placentas of preeclampsia patients; besides, autophagic levels are positively correlated with SBP and urine protein in patients with SPE.

## Introduction

Autophagy can be activated by various conditions, such as starvation, hypoxic environment, and high temperatures. LC3II is a reliable protein marker of autophagosome formation in mammalian cells, and the relative amount of LC3II reflects autophagic activity [1]. P62, also called SQSTM1, serving as a link between LC3 and ubiquitinated substrates, and P62-binding protein can be degraded in the autolysosomes, so it can be an index of autophagy [2].

Recent studies have uncovered that placental autophagy related to pathogenesis of preeclampsia and autophagic markers were found in cytotrophoblast, syncytiotrophoblast, and extravillous trophoblast [3].

The endoplasmic reticulum (ER), the major assembly site for almost all secretory and integral membrane proteins, is a vast membranous network. Unfolded or misfolded proteins accumulation in the ER gives rise to stress conditions.

The ER stress and autophagy, generally assumed as independent of each other and serving distinct functions, are involved in pathogenesis of preeclampsia [3, 4]. It is elucidated that ER stress response and autophagy are linked under some circumstances. These two systems are dramatically interconnected, and recent researches have demonstrated that ER stress can either stimulate or inhibit autophagy [5]. For example, a study by Le et al. [6] provided evidence that endogenous ER degradation-enhancing alpha mannosidase-like protein in nonstressed cells arrives in the cytosol that can be decomposed by basal autophagy. Periyasamy et al. [7] also reported when cocaine induces astrocytosis, ER stress can triggers autophagy. But on the other hand, some results support the view that diminished autophagy activity with aging contributes to increased adipose tissue ER stress and inflammation, means ER stress inhibit autophagy [8].

Urotensin II (UII) is a vasoactive substance, which is widely expressed in the placentas as well as in kidney, small intestine, and prostate [9, 10]. UII possesses the most potent endogenous vasoconstrictor discovered up to now [9].

Our previous study found that the expressions of UII and ERS markers (GRP78 and CHOP) were significantly higher in patients with severe preeclampsia (SPE) than that of normal controls. Moreover, these two were positively correlated [4]. According to previous study, we hypothesize that UII may induce placental autophagy through ER stress in SPE.

This paper is designed to explore the relationship between UII and autophagic markers in placentas of preeclampsia patients, and to illuminate the physiopathologic mechanism of preeclampsia.

## Materials and methods

### Study subjects

From 30 December 2015 to 30 November 2016, a total of 64 pregnant subjects were recruited, including 29 healthy pregnancies and 35 preeclamptic patients (7 mild preeclamptic patients and 28 severe preeclamptic patients). Consent forms were signed by all the participants.This study was supported by the Ethics Committee of Peking University Third Hospital.

Definition of SPE (one or more of following) [11]: systolic blood pressure (SBP) ≥ 160 mm Hg or diastolic blood pressure (DBP) ≥ 110 mm Hg on two occasions 6 or more hours apart in a pregnant woman on bed rest; proteinuria with excretion >2 g in 24 h urine; oliguria, with <500 ml over 24 h; pulmonary edema or cyanosis; impairment of liver function; visual or cerebral disturbances; pain in epigastric area or right upper quadrant; thrombocytopenia; and intrauterine growth restriction. Definition of mild preeclampsia (MPE): SBP ≥ 140 mm Hg or higher or DBP ≥ 90 mm Hg or higher coming up after 20 weeks’ gestation in a pregnant woman whose blood pressure was previously normal; and proteinuria with excretion of 0.3 g or more in a 24 h period.

The including criteria of preeclampsia patients were that patients must have pregnant-related hypertension and proteinuria, with a living fetus. The exclusive criteria in preeclamptic group were pregnancies with infection, twin pregnancies, diabetes mellitus, cancer, chronic liver disease, chronic heart disease, and chronic kidney disease. The control group was chosen at the same time admission for delivery in our hospital, and obtained consent form for their blood sample or placenta. Inclusive criteria for women in the control group were the participants delivery with single living fetus after 37 weeks, exclusive criteria for normal control were patients with hypertension, diabetes mellitus, primary liver disease, chronic kidney disease, or other diseases.

The way of delivery in normal control group was natural labor, and only five participants have done cesarean birth for scarred uterus; all the preeclpamtic patients have done cesarean birth.

Patients’ clinical data such as age, gestational age, height, body weight, etc. were collected. Body mass indices (BMIs) were calculated by weight before gestation divided by squared height. All participants’ blood pressure was measured by qualified physicians or nurses.

Venous blood samples were obtained from 29 healthy pregnancies and 35 preeclampsia patients in the fasting state before delivery. None of the patients were in labor at the time of sampling. Blood samples were collected and were immediately centrifuged before clotting. Serum were stored at −80 °C and were not thawed until analyzed. Hemoglobin, white cell counts, platelet counts, urine test strip, and biochemical analysis were measured with standard laboratory methods in our clinical laboratory. Twenty-four-hour urine was collected and urine protein quantitation was tested.

### UII measurement by radioimmunoassay

UII concentrations were measured by radioimmunoassay, and details about this assay can refer to our previous publications and other literature [12, 13].

### Immunohistochemical analyses for placentas

Immunohistochemical (IHC) analysis was utilized on placental tissues of 35 preeclamptic patients and 29 healthy normal control pregnancies. The paraffin-embedded placentas were sectioned at a thickness of 10 μm. Then, 5% hydrogen peroxide was incubated, following pre-treatment with 5% bovine serum albumin (BSA) for 30 min, the sections were performed with rabbit anti-human UII (1:1500, H-071-08, Phoenix Pharmaceuticals, Inc.), rabbit anti-human LC3 (1:200, ab48394, Abcam), and mouse anti-human P62 (1:100, ab56416, Abcam), phosphate-buffered saline as a substitute for the primary antibody at 4 °C overnight. The tissue slices were then incubated with second antibody (Zhongshan Gold Bridge Biotechnology Co. Ltd. Beijing, China) for 30 min, respectively. 3, 3′-diaminobenzidine staining in the dark was used to distinguish positive antigen from negative one. The sections were counterstained with hematoxylin. Brown deposits indicated positive staining. Then, 10 high-power microscope fields were randomly selected, and image pro plus 6.0 was used to calculate the integral optical density (IOD) of positive staining for UII, LC3, and P62 in placentas in normal controls, MPE patients, and SPE patients.

### Real-time PCR analysis for expression of UII, LC3, and P62 mRNA in placenta

UII, LC3, and P62 mRNA expression in placentas was analyzed by real-time PCR method. Total RNA was extracted using Trizol reagent (KeyGEN BioTECH, Nanjing, China). For first-strand cDNA synthesis, 1 µg RNA was reverse transcribed in a 20 µl reaction using Fast Quant RT Kit (TIANGEN, Beijing, China). Then, cDNAs were subjected to quantitative real-time PCR analysis.

For the UII primer sense 5′-CGTCTATCTTGTGGCGATCA-3′,

anti-sense 5′-CCCAGCATCTCTGGCAGTAT-3′;

LC3 primer sense 5′-GATGTCCGACTTATTCGAGAGC-3′,

anti-sense 5′-TTGAGCTGTAAGCGCCTTCTA-3′;

P62 primer sense 5′-GCACACCAAGCTCGCATTC-3′,

anti-sense 5′-ACCCGAAGTGTCCGTGTTTC-3′;

For glyceraldehyde 3-phosphate dehydrogenase (GAPDH) control prime sense

5′-GCGAGATCCCTCCAAAATCAA-3′,

anti-sense was 5′- GTTCACACCCATGACGAACAT-3′.

Real-time PCR was performed using an Quant Studio 5 Real-Time PCR System (Thermo Fisher Scientific, Waltham, MA, USA) in a 20 µl reaction consisting of 0.3 µM each primer, 100 ng template cDNA, 10 µl SuperReal Premix Plus with SYBR Green I (TIANGEN, Beijing, China), and 0.4 µl ROX reference dye. The PCR was run at 95 °C for 15 min followed by 40 cycles of 95 °C for 10 s and 60 °C for 32 s. All samples were analyzed in duplicate. GAPDH was used as an internal control. mRNA expression was calculated using the comparative threshold cycle (2^−△△CT^) method.

### Western blot analysis for placentas

Preeclampsia and healthy pregnancy proteins were extracted from the placentas, which were stored in liquid nitrogen. In short, the protein was first denatured at 100 °C for 5 min and then loaded on a SDS-polyacrylamide gel electrophoresis gel prior to being transferred to nitrocellulose membranes. Following blocked by 5% BSA, membranes were incubated with primary rabbit polyclonal anti-UII antibody (1:500; BS2918, Bioworld), primary rabbit polyclonal anti-LC3 antibody (1:500; ab48394, Abcam), and primary mouse monoclonal anti-P62 antibody (1 µg/ml; ab56416, Abcam) overnight at 4 °C, and then incubated with fluorescence-conjugated anti-rabbit and anti-mouse antibodies. Semiquantitative grayscale intensity was measured by ImageJ.

### Statistical analysis

Data are presented as means ± standard deviation. Independent sample *T* test or one-way analysis of variance (ANOVA) test was applied in statistical analysis. Least significant difference (variance similar) or Dunnett’s t3 (variance without similar) was performed for post hoc analysis after we used ANOVA. Pearson correlation coefficients were calculated to evaluate the relationship between the expression of UII and autophagic markers. Mann-Whitney *U* test was applied to test the difference for non-normal distribution. Non-normal distributed data were normalized to its natural logarithm.The data were analyzed using the statistical package SPSS 17.0 (SPSS, Inc., Chicago, IL, USA). A two-sided *P* < 0.05 was considered statistically significant.

## Results

### Clinical characteristics of participants

Table 1 summarized the clinical characteristics and biochemical data of all participants. Obviously, preeclamptic patients had higher levels of BMI, SBP, DBP, and 24 h urine protein quantitation than those of normal controls (*P* < 0.05). Moreover, there were significantly higher urinary protein quantitative level and significantly lower gestational weeks in SPE patients compared to MPE patients (*P* < 0.05). Besides, circulating UII is significantly higher in preeclamptic patients compared to normal controls. There were no significant differences in age, white blood cell, hemoglobin, and urinary UII between preeclamptic patients and normal pregnancy.

**Table 1:** Comparison of clinical parameters between preeclampsia group and normal controls (*n* = 64)

Index	Normal control (*n* = 29)	MPE (*n* = 7)	SPE (*n* = 28)
Age (years)	31.7 ± 3.9	32.3 ± 4.7	31.5 ± 5.9
BMI (kg m^−2^)	27.6 ± 3.1	30.8 ± 4.9*	29.6± 4.5*
GA (weeks)	38.4 ± 2.7	36.7 ± 2.6	34.0 ± 2.2*^#^
SBP (mm Hg)	116.9 ± 9.4	139.9 ± 20.1*	146.7 ± 17.9*
DBP (mm Hg)	73.8 ± 8.6	90.8 ± 13.0*	89.2 ± 12.8*
Urinary protein level (g per 24 h)		1.005 (0.317, 1.987)*	4.510 (2.145, 20.013)*^#^
WBC (×10^9^ l^−1^)	8.69 ± 1.81	10.17 ± 1.87	11.43 ± 2.15
Hb (g l^−1^)	115.6 ± 13.7	112.9 ± 16.1	116.2 ± 18.7
Serum UII (pg ml^−1^)	22.1 ± 12.20	32.13 ± 14.90	43.69 ± 21.13*
Urine UII (pg ml^−1^)	11.35 (7.19, 20.13)	12.89 (8.61, 22.67)	16.11 (9.02, 22.49)

*P* values below 0.05 have been accepted to be significant. Normal distribution data are presented as means ± s.d. Abnormal distribution data are presented as median, 25 percentile, and 75 percentile

*MPE* mild preeclampsia, *SPE* severe preeclampsia, *BMI* body mass index, *GA* gestational age, *SBP* systolic blood pressure, *DBP* diastolic blood pressure, *WBC* white blood cell, *Hb* hemoglobin, *UII* urotensin II

*P < 0.05 compared with normal controls, ^**#**^*P* < 0.05 compared with MEP group

### Immunohistochemical analysis of expression levels of UII, LC3, and P62 in placental tissue

**Fig. 1:**
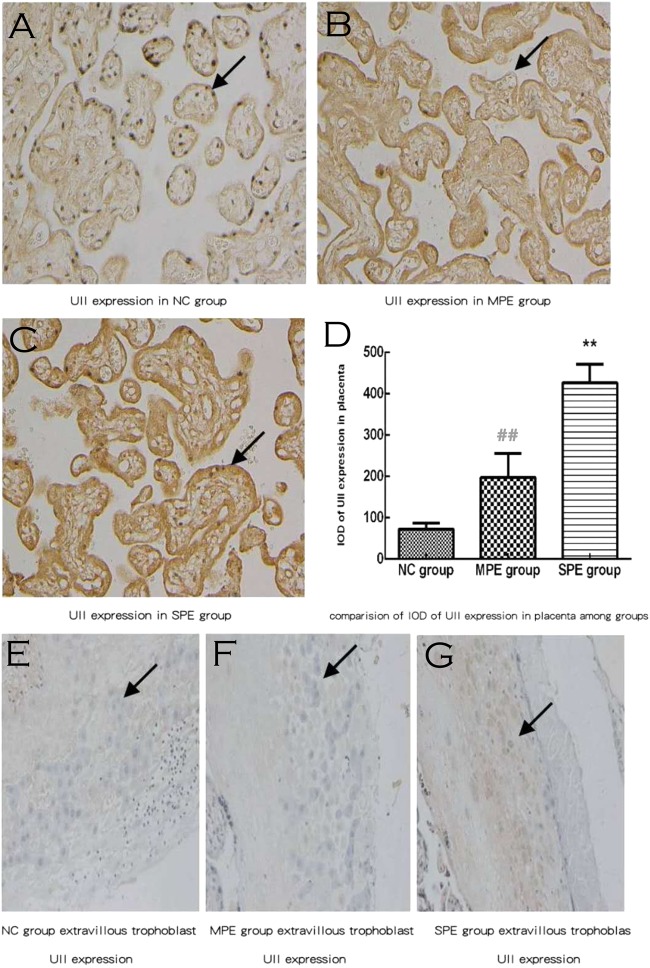
Expressions of UII in placental tissue by immunochemistry. Expressions of UII in SPE group were highly increased and were mainly located in the cytoplasm of placental trophoblastic cells and syncytiotrophoblast cells (brown deposits) **a** for normal pregnancy; **b** for MPE group; and **c** for SPE group. **e** For NC group, **f** MPE group, and **g** SPE group, UII expression was located in extravillous trophoblast (brown deposits). There was significantly higher integral optical density (IOD) of UII expression in placenta in SPE group in comparison with normal controls and MPE group by semiquantitative analysis (**d**) (**P < 0.01 compared with normal control; ^**##**^*P* < 0.01 compared with SPE group). NC normal control (color figure online)

UII was located mainly in the cytoplasm of placental cytotrophoblastic cells and syncytiotrophoblast cells (brown deposits; Fig. 1a–c). The expression of UII of SPE patients was higher than that of MPE patients and normal controls. UII expression was also located in extravillous trophoblast (brown deposits; Fig. 1e–g). There was significantly higher IOD of UII expression in placentas in SPE in comparison to normal controls and MPE group by semiquantitative analysis (Fig. 1d), whereas there was no difference in IOD of UII expression in placentas in MPE group in comparison to normal controls by semiquantitative analysis (*P* > 0.05).

**Fig. 2:**
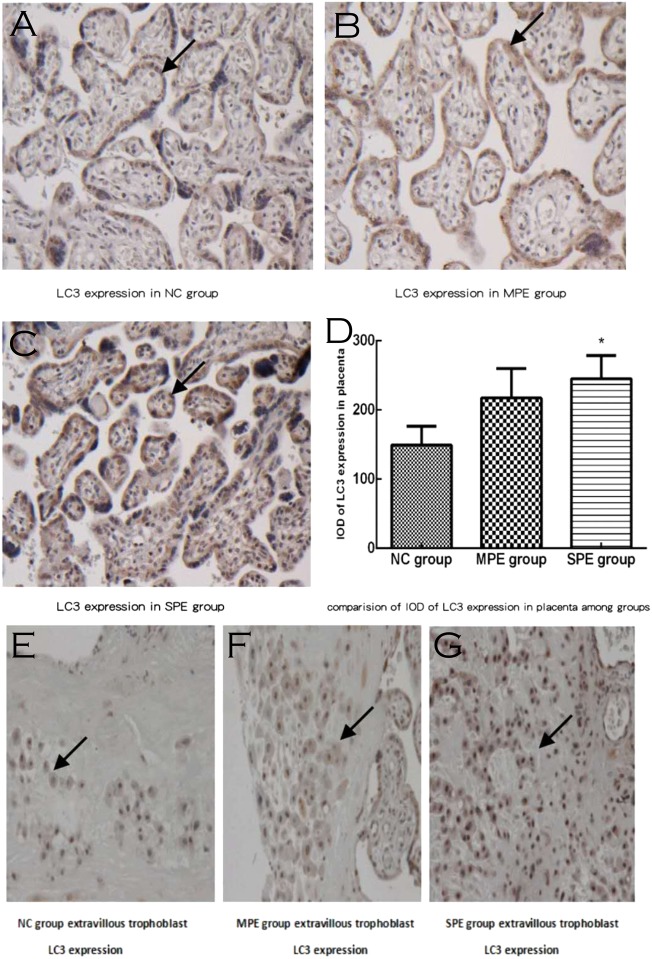
Expressions of LC3 in placental tissue by immunochemistry. Expressions of LC3 in SPE group were highly increased and were mainly located in the cytoplasm of placental trophoblastic cells and syncytiotrophoblast cells (brown deposits), **a** for normal pregnancy; **b** for MPE group; and **c** for SPE group. **e** For NC group, **f** MPE group, and **g** SPE group, LC3 expression was located in extravillous trophoblast (brown deposits). There was significantly higher integral optical density (IOD) of LC3 expression in placenta in SPE group in comparison with normal controls by semiquantitative analysis (**d**) (*P < 0.05 compared with normal control) (color figure online)

LC3 expressions were located in placental cytotrophoblastic cells and syncytiotrophoblast cells (brown deposits; Fig. 2a–c). LC3 expressions were also existed in extravillous trophoblast (brown deposits; Fig. 2e–g). In our current study, we mainly collected the placenta tissue from fetal side not the placenta tissue from maternal side, so it was difficult to find the extravillous trophoblast, so we only analyzed cytotrophoblast and syncytiotrophoblast autophagy markers by immunochemistry or western blot.

There was significantly higher IOD of LC3 expression in placentas in SPE in comparison to normal control by semiquantitative analysis (245.08 ± 142.55 vs. 149.43 ± 94.14, *P* = 0.044; Fig. 2d), whereas there was no difference in IOD of LC3 expression in placentas in MPE in comparison to normal controls and SPE group by semiquantitative analysis (*P* > 0.05).

**Fig. 3:**
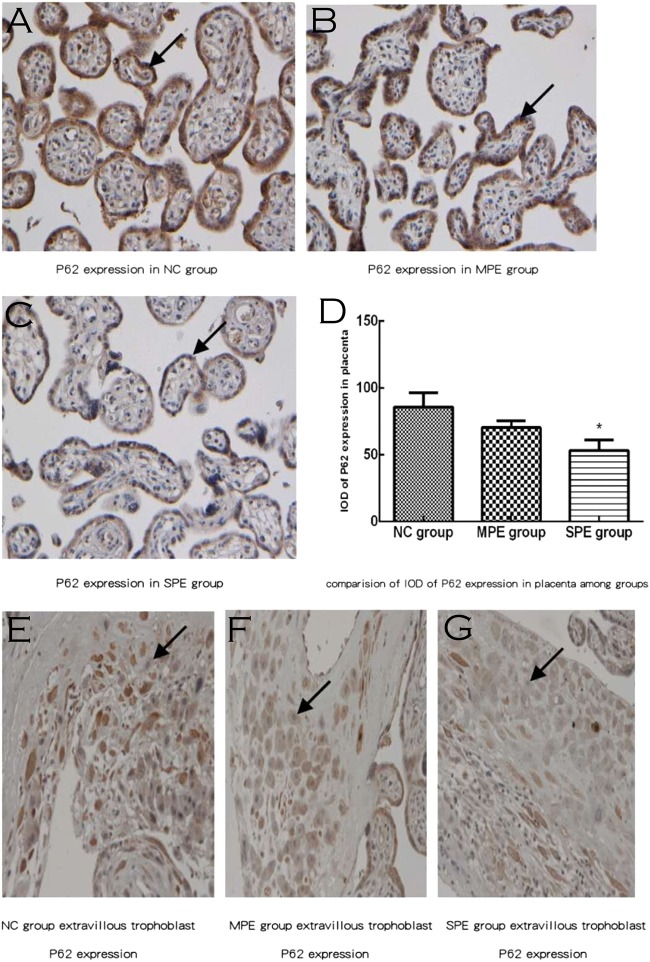
Expressions of P62 in placental tissue among normal control (NC), MPE group, and SPE group by immunochemistry. Expressions of P62 in SPE group were highly decreased and were mainly located in the cytoplasm of placental trophoblastic cells and syncytiotrophoblast cells (brown deposits) **a** for normal pregnancy; **b** for MPE group; and **c** for SPE group. **e** for NC group, **f** MPE group, and **g** SPE group, P62 expression was located in extravillous trophoblast (brown deposits) There was significantly lower integral optical density(IOD) of P62 expression in placenta in SPE group in comparison to normal control group by semiquantitative analysis(*P < 0.05 compared with NC) (color figure online)

P62 expressions were existed in placental cytotrophoblastic cells and syncytiotrophoblast cells (brown deposits; Fig. 3a–c). P62 expressions were also located in extravillous trophoblast (brown deposits; Fig. 3e–g). There was significantly lower IOD of P62 expression in placenta in SPE in comparison to normal control by semiquantitative analysis, whereas there was no difference in IOD of P62 expression in placenta in MPE group in comparison to normal control and SPE group by semiquantitative analysis (*P* > 0.05; Fig. 3d).

### Comparison of mRNA expressions of UII and autophagic markers in placental tissue between preeclamptic patients and normal pregnancy by real-time quantitative PCR

**Fig. 4:**
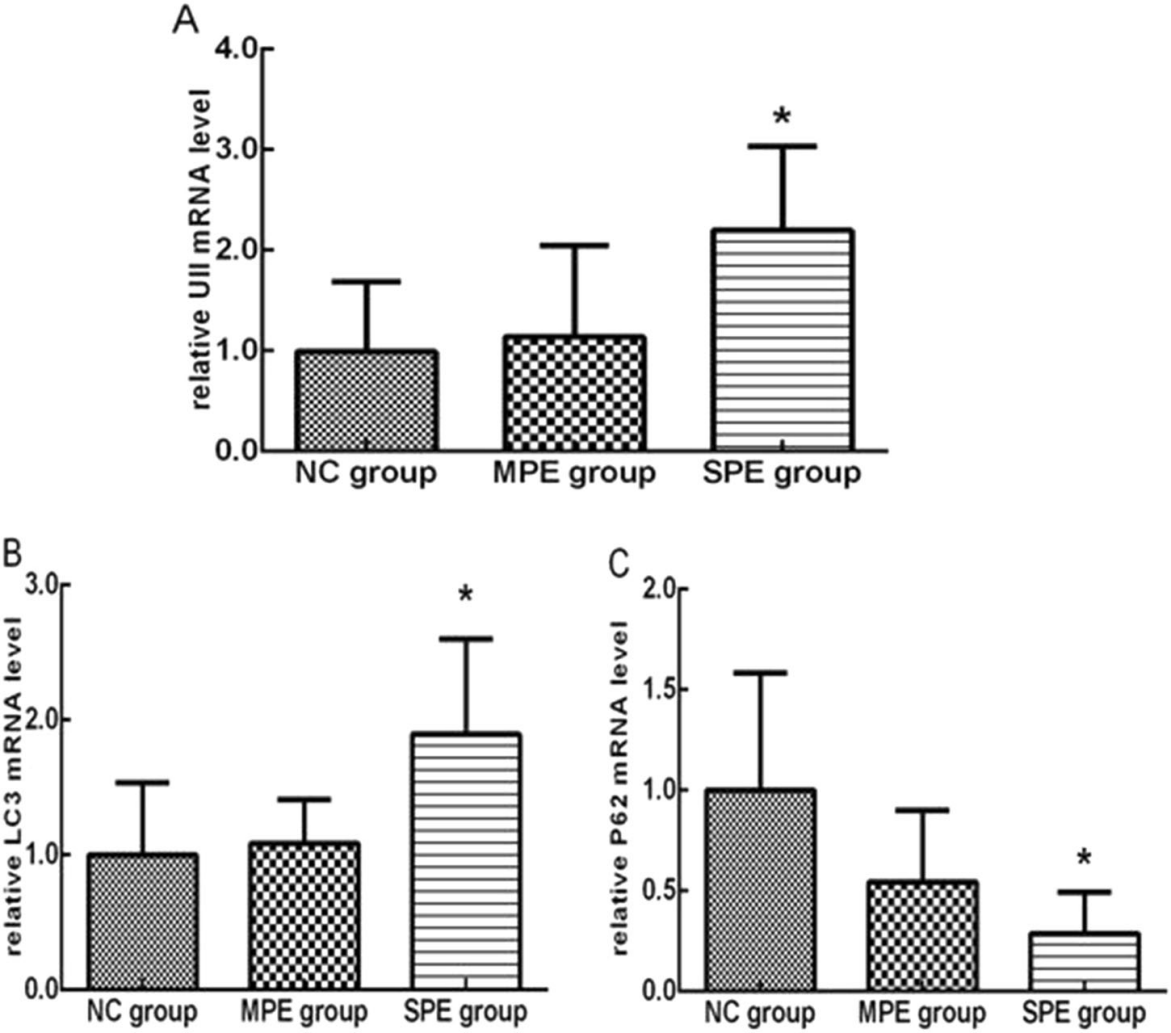
mRNA expressions of *UII* (**a**), *LC3* (**b**), and *p62* (**c**) in placentas among normal control (NC), MPE group, and SPE group by real-time PCR (*P < 0.05 compared with NC)

Real-time quantitative PCR analysis showed that expression of *UII* mRNA was significantly higher in SPE group in comparison to normal controls (2.20 ± 0.77 vs. 0.99 ± 0.64, *P* < 0.05; Fig. 4a). However, there was no difference in *UII* mRNA expression of MPE group in comparison to normal controls and SPE group (*P* > 0.05; Fig. 4a).

mRNA expression of* LC3* was significantly higher in SPE group in comparison to normal controls (Fig. 4b) (1.89 ± 0.65 vs. 1.00 ± 0.48, *P* < 0.05), but there was no significant difference in the expression of *LC3* gene of MPE group in comparison to normal controls and SPE group (*P* > 0.05). *P62* mRNA expression was significantly lower in SPE group in comparison to normal group (0.29 ± 0.19 vs. 1.00 ± 0.53, *P* < 0.05), but there was no significant difference in MPE patients in comparison to normal controls and SPE group (*P* > 0.05; Fig. 4c).

### Comparison of protein expressions of UII and autophagic markers in placental tissue between preeclamptic patients and normal pregnancy by western blot

**Fig. 5:**
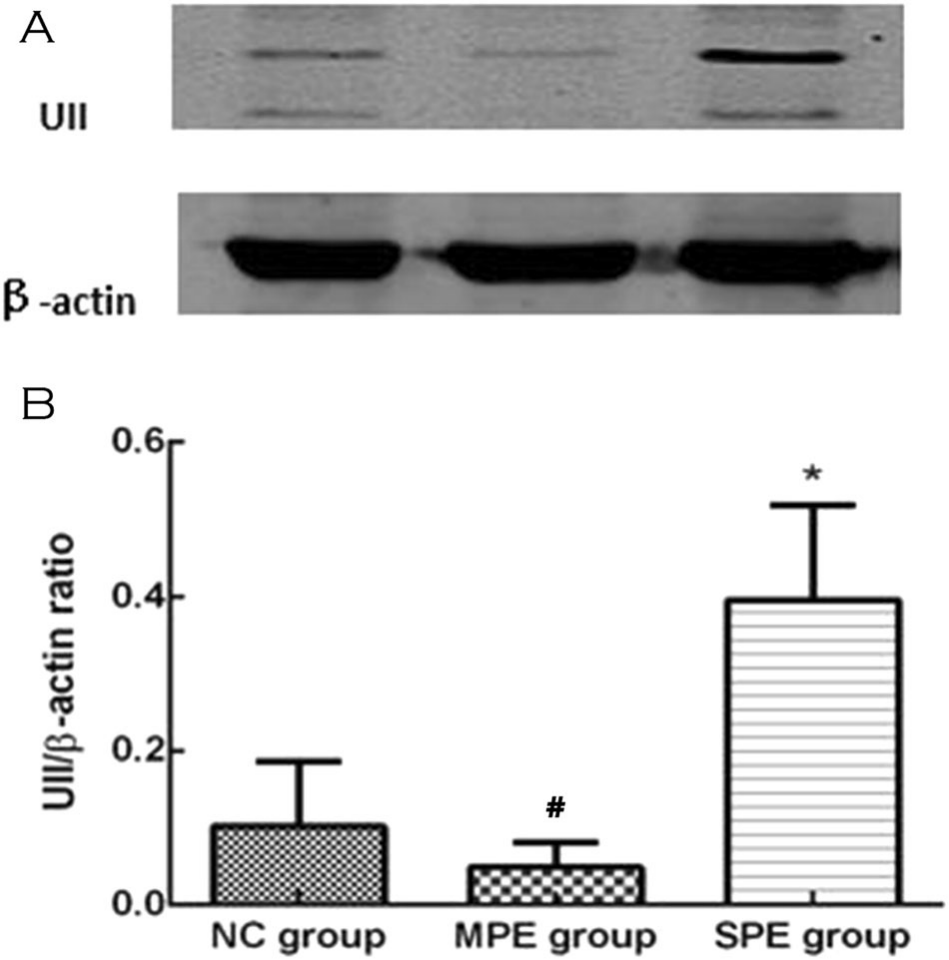
Protein expressions of UII in placentas with preeclampsia and normal control (NC). There were significantly higher protein expressions of UII in placentas with SPE compared with NC (*P < 0.05 compared with NC), and there was significant difference between MPE group and SPE group (^**#**^*P* < 0.05 compared with SPE group)

Western blot analysis showed that UII protein expression was significantly higher in SPE group in comparison to MPE group and normal controls, but there was no difference between MPE group and NC group (Fig. 5).

**Fig. 6:**
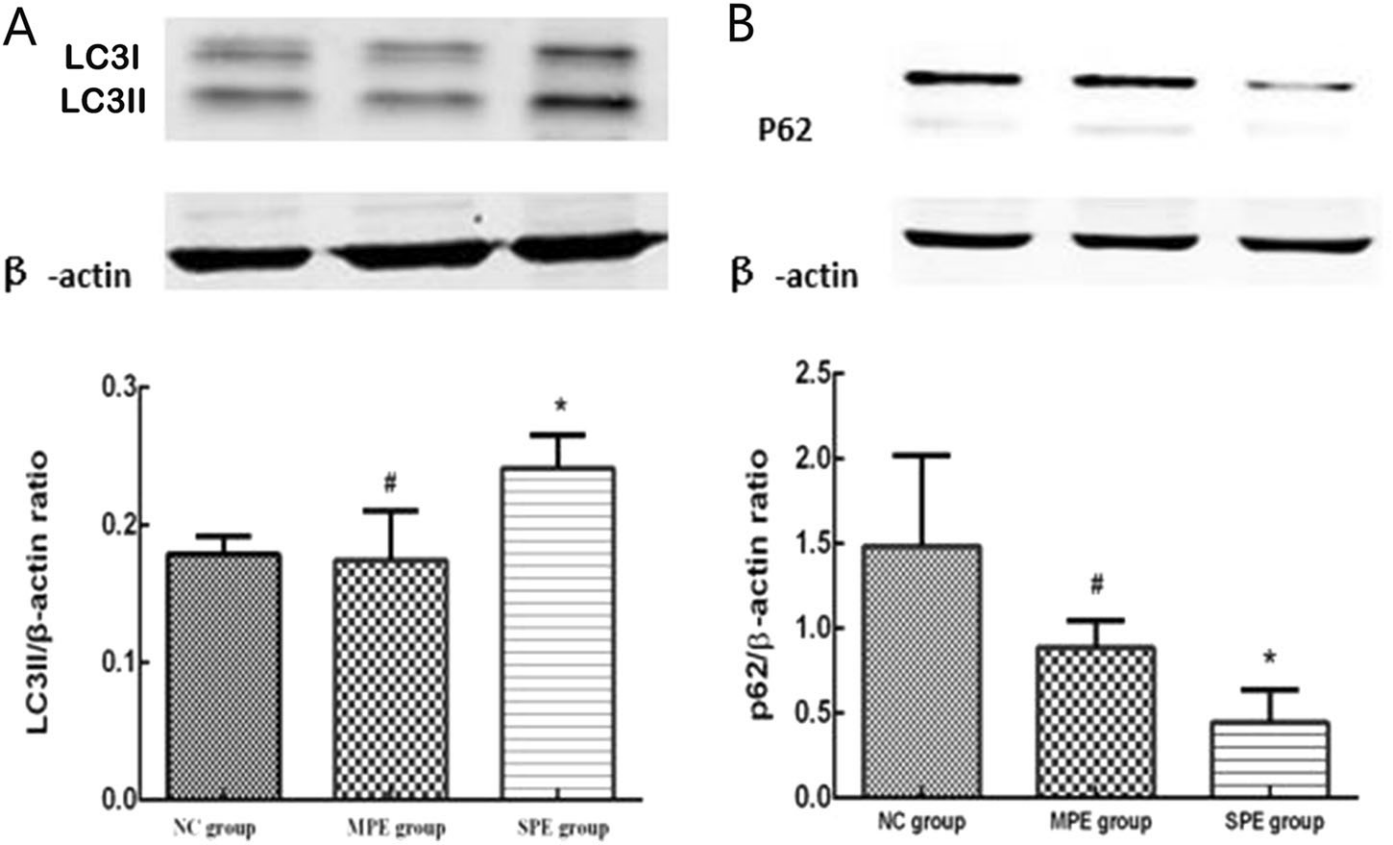
Protein expressions of LC3II in placentas with preeclampsia and normal control (NC) (**a**). Moreover, the expression of LC3II was significantly higher in SPE group compared with normal controls and MPE group (*P < 0.05 compared with NC; ^**#**^*P* < 0.05 compared with SPE group). Protein expressions of P62 in placentas with preeclampsia and normal control (NC) (**b**). P62 expression was significantly lower in SPE group in comparison to normal ones and MPE group (*P < 0.05 compared with NC; ^**#**^*P* < 0.05 compared with SPE group)

Western blot demonstrated that there existed the expressions of LC3II and P62 in placentas of pregnancies (Fig. 6a, b). Moreover, the expression of LC3II was significantly higher in SPE group compared with normal controls and MPE group (*P* < 0.05), but there was no significant difference in the expression of LC3II between normal controls and MPE group (*P* > 0.05). P62 expression was significantly lower in preeclampsia group, MPE group, and SPE group in comparison to normal ones (*P* < 0.05), but there was no significant difference between SPE group and MPE group (*P* > 0.05).

### Correlation between protein expression UII and autophagic markers (LC3II and P62) in placenta of patients with preeclampsia and their relationships with other parameters

The IOD of UII by IHC was positively correlated with SBP (*r* = 0.614, *P* < 0.001), DBP (*r* = 0.450, *P* = 0.003), and blood uric acid (*r* = 0.592, *P* = 0.008), but had no correlation with blood creatinine, blood urea nitrogen, and urine protein (Table 2). Western blot analysis showed that UII expression was positively related to SBP (*r* = 0.489, *P* = 0.015) and urine protein quantitation (*r* = 0.784, *P* = 0.004; Table 3).

**Table 2:** The correlation of IOD of UII and autophagic markers (LC3II and P62) and their relationships with other parameters

Variables	UII	LC3II	p62
SBP (mm Hg)	*r* = 0.614, *P* < 0.001	*r* = 0.501, *P* = 0.003	*r* = −0.419, *P* = 0.024
DBP (mm Hg)	*r* = 0.450, *P* = 0.003	*r* = 0.217, *P* = 0.225	*r* = −0.492, *P* = 0.006
Urine protein levels (g per 24 h) (preeclampsia group)	*r* = 0.117, *P* = 0.605	*r* = 0.509, *P* = 0.022	*r* = −0.594, *P* = 0.020
BUN (mmol l^−1^)	*r* = 0.156, *P* = 0.392	*r* = 0.081, *P* = 0.705	*r* = 0.001, *P* = 0.995
Blood creatinine (μmol l^−1^)	*r* = 0.177, *P* = 0.334	*r* = 0.361, *P* = 0.083	*r* = 0.263, *P* = 0.237
Blood UA (μmol l^−1^)	*r* = 0.592, *P* = 0.008	*r* = 0.355, *P* = 0.195	*r* = −0.099, *P* = 0.726
UII		*r* = 0.495, *P* = 0.010	*r* = 0.164, *P* = 0.502

*SBP* systolic blood pressure, *DBP* diastolic blood pressure, *BUN* blood urea nitrogen, *UA* uric acid, *UII* urotensin II

*P* values below 0.05 have been accepted to be significant. *P < 0.05 compared with normal controls; ^**#**^*P* < 0.05 compared with MEP group

**Table 3:** The correlation between UII and autophagic markers (LC3II and P62) by western blot and their relationships with other parameters

Variables	UII	LC3II	p62
SBP (mm Hg)	*r* = 0.489, *P* = 0.015	*r* = 0.623, *P* = 0.073	*r* = −0.478, *P* = 0.098
DBP (mm Hg)	*r* = 0.299, *P* = 0.244	*r* = 0.618, *P* = 0.076	*r* = −0.302, *P* = 0.315
Urine protein levels (g per 24 h) (preeclampsia group)	*r* = 0.784, *P* = 0.004	*r* = 0.601, *P* = 0.207	*r* = 0.376, *P* = 0.406
BUN (mmol l^−1^)	*r* = 0.346, *P* = 0.270	*r* = 0.493, *P* = 0.320	*r* = −0.267, *P* = 0.523
Blood creatinine (μmol l^−1^)	*r* = 0.308, *P* = 0.330	*r* = 0.352, *P* = 0.493	*r* = −0.114, *P* = 0.788
Blood UA (μmol l^−1^)	*r* = 0.051, *P* = 0.904	*r* = 0.888, *P* = 0.304	*r* = −0.969, *P* = 0.031
UII		*r* = 0.816, *P* = 0.007	*r* = −0.448, *P* = 0.116

*SBP* systolic blood pressure, *DBP* diastolic blood pressure, *BUN* blood urea nitrogen, *UA* uric acid, *UII* urotensin II

*P* values below 0.05 have been accepted to be significant. *P < 0.05 compared with normal controls; ^**#**^*P* < 0.05 compared with MEP group.

IHC analysis revealed that the IOD of LC3 was positively correlated with SBP (*r* = 0.501, *P* = 0.003) and urine protein quantitation (*r* = 0.509, *P* = 0.022), whereas the expression of P62 has negative correlation with SBP (*r* = −0.419, *P* = 0.024), DBP (*r* = −0.492, *P* = 0.006), and urine protein quantitation (*r* = −0.594, *P* = 0.020), but both of them had no correlation with blood creatinine (Table 2). Western blot analysis showed that neither of them had correlation with blood pressure and urine protein (Table 3).

Western blot and IHC analysis both indicate that the expression of UII was significantly correlated with autophagic marker  LC3 (*r* = 0.495, *P* = 0.010; *r* = 0.816, *P* = 0.007), whereas had no correlation with P62 (Table 3).

## Discussion

Autophagy is activated under some starvation or hypoxic conditions and it is also another way of cell death, that is, autophagic programmed cell death [14]. The underlying pathogenesis of preeclampsia hasn’t been clearly identified at present. Recent studies have revealed that placental autophagy correlates with pathogenesis of preeclampsia [3, 15–19].

Oh et al. [15] confirmed LC3II was upregulated in preeclampsia group (11 subjects) in comparison to normal controls (8 subjects). Moreover, he found autophagosomes in placentas of SPE patients by electron microscope. Gao et al. [16] observed autophagosome precursors in the syncytiotrophoblast and vessel endothelial cells by electron microscope. He confirmed that LC3 and Beclin-1 expression in the syncytiotrophoblast and vessel endothelial cells of placentas of patients with early-onset preeclampsia (<34 gestational weeks). Moreover, the expression of LC3 and Beclin-1 was highly increased in early-onset preeclampsia group compared to normal controls. Autophagy activation was also reported in preeclamptic placentas and fetal growth restriction [17]. Autophagic vacuoles were observed on electron micrographs in the syncytiotrophoblast layer of human placentas [17, 18], and autophagic vacuoles were seen more in fetal growth restriction [17]_._ Moreover, Melland-Smith et al. [19] recently elucidated that the combination of ceramide-mediated autophagy activation and oxidative stress-reduced hydrolase activity impaired placental function in preeclampsia which were accompanied by a reduction in N-acylsphingosine amidohydrolase 1, which catalyzes the degradation of ceramide into sphingosine and free fatty acid.

Our study confirmed that there actually existed the expression of LC3 and P62 in cytoplasm of trophoblast in placenta by IHC. Moreover, IHC analysis and western blot analysis both demonstrated there was significantly higher expression of LC3 and significantly lower expression of P62 in placenta of SPE group than that of normal control group, which suggested autophagy was upregulated in placenta of patients with SPE. This was consistent with most studies [3, 15–19]. Moreover, in our current study, we found there was significant different autophagy level between SPE group and MPE group by western blot, but there was no difference in autophagy level between MPE and normal control. This may mean there was different pathogenesis between SPE and MPE group.

The placental autophagic markers were found in cytotrophoblast, syncytiotrophoblast, and extravillous trophoblast, but we mainly collected the placenta tissue from fetal side not the placenta tissue from maternal side in our current study, so it was difficult to find the extravillous trophoblast; we provide some pictures for UII, LC3, and P62 expression in extravillous trophoblast (Figs. 1e–g, 2e–g, 3e–g) but they are very few, so we only analyzed cytotrophoblast and syncytiotrophoblast autophagy markers by immunochemistry or western blot in our current investigation.

Our study first demonstrated that LC3 expression in placentas of pregnancies by IHC or western blot was positively correlated with blood pressure, whereas P62 was negatively correlated with blood pressure. It might result from placenta’s trophoblast and endothelial cell overactivation, which affects trophoblast invasion and consequently regulates blood pressure by placentas releasing active vascular substances; on the other hand, placenta’s endothelial cell autophagy induces vascular remodeling and vascular constricting, and then increases blood pressure.

Moreover, we first elucidate that autophagic levels are positively correlated with urine protein level in patients with SPE. Some researchers reported that autophagy was correlated with proteinuria in diabetic nephropathy. Wang et al. [20] recently demonstrated that diabetic rats treated with mangiferin for a period had dramatically decreased albuminuria and glomerular extracellular matrix expansion and more podocyte marker expression; moreover, mangiferin delayed the progression of diabetic nephropathy and protected the podocytes by enhancing autophagy under diabetic conditions. In our current study, we first verify that placenta’s autophagy levels are positively correlated with proteinuria. Two reasons might account for this phenomenon. First, placenta’s autophagy overactivation could induce progressive preeclampsia; consequently, placental tissue releases some active cytokines or some active substances to circulation, which long distance induce the injury of glomeruli endothelial and increase proteinuria. Second, we also found placenta’s UII expression is positively correlated with proteinuira. We speculate that UII has harmful effect in preeclampsia (consisting of preeclampsia kidney lesion) through enhancing autophagy of trophosblast. Next step, we need to elucidate whether UII induce autopahgy of trophosblast in in vitro study and in animal experiment.

Preeclampsia pathogenesis involved ER stress and autophagy [3, 4]. Previous studies have suggested that elevated Ca^2+^concentration occurring in ER stress could induce development of autophagy [5]. Our previous study also confirmed that the UII expression had positive correlation with ER stress markers GRP78 and CHOP, blood pressure, and urine protein in patients with preeclampsia [4]. In view of these, we speculated that UII might have correlation with autophagy of placenta. In our current investigation, we verify that UII is positively correlated with placental autophagy. These hints under hypoxia condition, placental tissue releases UII and UII may promote autophagic level of placentas, and then induces and aggravates preeclampsia through ER stress pathway in preeclampsia.

There are some limitations in our current study. In pathogenetic perspective, initiation of preeclampsia(PE) pathology indeed occurs in the first trimester. Nevertheless, many patients with early preeclampsia can successfully continue pregnancy after they are managed carefully by obstetrician, cardiologists, and nephrologists until 32 weeks. On the other hand, some patients terminate pregnancy before 28 weeks so that it is difficult to collect these patients’ data and placentas, so we conducted the study in late-trimester preeclampsia patients.

## Conclusion

Our study certifies that UII expression and autophagy are significantly upregulated in placentas of patients with preeclampsia. We first verify both UII and autophagy in placentas are positively correlated with SBP and urine protein; besides, UII also has positive correlation with autohagy in placentas of preeclampsia patients. This indicates that UII may be implicated in the development of preeclampsia by promoting autophagy of trephocyte.

### Summary Table

#### What is known about topic?

- Upregulation of urotensin II is associated with upregulation of autophagy in placenta of patients with severe preeclampsia.

#### What this study adds?

1. We first verify both UII and autophagy in placentas are positively correlated with systolic blood pressure and urinary protein.
2. UII also has positive correlation with autophagy in placentas of preeclampsia patients, hinting that UII may be participated in the development of preeclampsia by promoting autophagy of trephocyte.
